# The Digital Mirror: Clinical Potentials and Relational Risks of Generative AI in Mental Health Interventions

**DOI:** 10.1007/s11920-026-01690-4

**Published:** 2026-06-18

**Authors:** Cesare Cavalera, Fabio Frisone, Chiara Rossi, Osmano Oasi, Francesco Pagnini, Giuseppe Riva, Lorenzo Antichi

**Affiliations:** 1 Department of Psychology, Università Cattolica del Sacro Cuore, Via Nirone 15, Milan, 20123 Italy; 2 Sophia University Institute, Firenze, Italy; 3 Department of Human Science, Università degli Studi Guglielmo Marconi, Rome, Italy; 4 Humane Technology Laboratory, Università Cattolica del Sacro Cuore, Milan, Italy; 5 Applied Technology for Neuro-Psychology Laboratory, Istituto Auxologico Italiano IRCCS, Milan, Italy

**Keywords:** Generative Artificial Intelligence, Digital Mental Health, Chatbot-Delivered Interventions, Psychotherapy Process, Natural Language Processing, Ethics in AI

## Abstract

**Purpose of Review:**

This review explores the rapidly evolving integration of Generative Artificial Intelligence (GenAI) in mental health care. It aims to evaluate current applications in assessment, treatment planning, and psychotherapeutic interventions, while critically examining the clinical risks, ethical dilemmas, and the future potential of GenAI as an adjunctive tool rather than a replacement for human-delivered therapy.

**Recent Findings:**

Recent studies indicate that AI models can effectively assist in diagnostic reasoning, biomarker identification via EEG, and the prediction of symptom trajectories from session transcripts. Randomized controlled trials (RCTs) suggest that GenAI chatbots significantly reduce anxiety and depressive symptoms in the short term, particularly in settings with limited access to clinicians. However, human-led therapy remains superior in fostering deep emotional engagement and clinical impact. Significant risks identified include the potential for GenAI to foster dependency, reinforce maladaptive schemas or delusional ideation through “sycophantic” mirroring, and raise complex ethical-legal challenges regarding the reporting of criminal disclosures.

**Summary:**

AI represents a transformative adjunctive layer in mental health, offering scalable support for assessment, training, and between-session monitoring. While technological advances in personalization, multimodality, and immersive virtual reality enhance its clinical utility, GenAI lacks the authentic relational depth and"calibrated mismatches" essential for autonomy and transformative change. Future integration must prioritize a human-centered, blended approach, where GenAI is strictly supervised by clinicians within a robust ethical and regulatory framework to preserve the essential heart of the therapeutic connection. Research priorities, interim clinical safeguards, and recommendations for navigating the gap between current evidence and real-world adoption need to be defined and implemented.

## Introduction

The current gap between demand and available services underscores the need to scale up psychotherapeutic interventions while preserving accessibility, quality, and personalization [1]. In this context, the integration of Generative Artificial Intelligence (GenAI) into clinical practice is beginning to reshape approaches to mental health care. GenAI refers to a class of AI systems that can create new content, such as text, dialogue, images, audio, or personalized feedback, by learning patterns from large datasets. In mental health field, GenAI may be applied to power conversational agents, draft psychoeducational material, summarize clinical notes, simulate therapeutic dialogue, or provide tailored self-help responses.

Other forms of AI in mental health are typically oriented towards analysis, prediction, classification, or decision support rather than content generation. For example, machine learning (ML) models may predict risk of relapse, classify depression severity from speech or questionnaire data, detect patterns in electronic health records, or support diagnosis and treatment planning. The key difference is therefore primarily functional: GenAI produces new, human-like content, while traditional or non-generative AI mainly identifies patterns, makes predictions, or supports decisions based on existing data. In mental health field, this distinction is important because GenAI directly interacts with language, meaning, and therapeutic communication, whereas other AI systems more often operate as assessment, monitoring, or clinical support tools. Recent advances in ML and natural language processing (NLP) enable the analysis of multimodal data sources, including self-report measures, digital phenotyping, electronic health records (EHRs), speech features, smartphone use, and other passive data streams. Early evidence suggests that AI may support diagnostic processes, treatment planning, and symptom-focused interventions [2, 3]. Moreover, the integration of large knowledge bases with Retrieval Augmented Generation (RAG) enables virtual therapists to deliver structured psychoeducational content efficiently [4]. Combined with relatively low access costs, these features have contributed to the growing diffusion of virtual therapists, raising the prospect of broader access to care [5]. At the same time, several studies highlight important limitations, particularly in complex clinical presentations, where communication difficulties and reduced empathic depth remain evident [6–8]. Persistent concerns also remain regarding data storage, privacy, and cybersecurity risks in cloud-based healthcare systems.

In the wake of these innovations, psychotherapists face an exciting challenge: to clearly reaffirm the core elements of their professional identity while thoughtfully exploring the potential role of AI in therapy. Hence, the present narrative review provides a careful look at the current data on AI applications across key areas: (1) assessment and treatment planning, (2) psychotherapeutic intervention, (3) clinical risks, (4) future integration, and (5) recommendations for research, clinical practice, and the evidence–adoption gap.

## Methods

We conducted a narrative review of key AI applications in mental health over the past fifteen years, emphasizing studies with clear clinical relevance and prioritizing those that examined GenAI use directly within clinical settings. We have included brief descriptions of key AI terminology (Table 1) and key takeaways related to the different domains (Table 2).

**Table 1:** AI psychotherapist keywords Key terminology

Term	Operational definition	Includes	Excludes	References
AI chatbot	A system designed to interact with users via natural language, rule-based or AI-driven.	Rule-based bots; AI chatbots delivering exercises or psychoeducation.	A licensed therapist or autonomous clinical agent.	Rane, A., Ranade, C., Bandekar, H., Jadhav, R., & Chitre, V. (2022, December). AI driven Chatbot and its Evolution. In *2022 5th international conference on advances in science and technology (ICAST)* (pp. 170–173). IEEE.
AI-enabled clinical decision support	AI systems designed to support (not replace) clinician judgment in assessment and treatment planning.	Screening flags; summaries; pattern detection; draft formulations.	Autonomous diagnosis or treatment decisions.	Elhaddad, M., & Hamam, S. (2024). AI-driven clinical decision support systems: an ongoing pursuit of potential. *Cureus*, *16*(4), e57728.
Anthropomorphization	Users’ tendency to attribute understanding or intentionality to computational systems.	Perceived empathy; emotional bonding with AI.	Evidence of clinical efficacy or insight.	Kim, Y., & Sundar, S. S. (2012). Anthropomorphism of computers: Is it mindful or mindless?. *Computers in Human Behavior*, *28*(1), 241–250.
Automation bias	Over-reliance on automated system outputs, leading to reduced critical judgment.	Clinician or user over-trust in AI recommendations.	Bias eliminated by high system accuracy alone.	Cummings, M. L. (2017). Automation bias in intelligent time critical decision support systems. In *Decision making in aviation*(pp. 289–294). Routledge.
Blended approach	A structured integration of human psychotherapy with digital or AI-based components supporting treatment.	Homework apps, monitoring tools, digital between-session support.	Replacement of the clinician or delegation of clinical risk.	Wentzel, J., Van der Vaart, R., Bohlmeijer, E. T., & van Gemert-Pijnen, J. E. (2016). Mixing online and face-to-face therapy: how to benefit from blended care in mental health care. *JMIR mental health*, *3*(1), e9.
Conversational agent	A conversational system explicitly designed to establish rapport and long-term engagement with users.	Embodied agents; continuity of interaction; affective language.	Psychotherapy or clinical competence per se.	Bickmore, T., & Gruber, A. (2010). Relational agents in clinical psychiatry. *Harvard review of psychiatry*, *18*(2), 119–130.
Digital mental health intervention	A broad category of mental health–oriented interventions delivered via digital technologies, not necessarily involving psychotherapy	Apps, iCBT programs, psychoeducation, symptom tracking.	Automatic equivalence with psychotherapy or a “therapist.”	Pineda, B. S., Mejia, R., Qin, Y., Martinez, J., Delgadillo, L. G., & Muñoz, R. F. (2023). Updated taxonomy of digital mental health interventions: a conceptual framework. *Mhealth*, *9*, 28.
Digital phenotyping	In situ quantification of behavior and experience using data from personal digital devices.	Smartphone sensors; wearables; Ecological Momentary Assessment + passive data.	Stand-alone diagnosis or treatment decisions.	Marsch, L. A. (2018). Opportunities and needs in digital phenotyping. *Neuropsychopharmacology*, *43*(8), 1637–1638.
Generative AI (GenAI) / Large Language Model (LLM)	A probabilistic model that generates language based on training data and prompts.	Text generation; conversational interfaces; summarization.	Intentional understanding; guaranteed factual accuracy.	Frisone, F., Pupillo, C., Rossi, C., & Riva, G. (2026). Toward Clinical Integration of Generative AI in Mental Health: Personalization, Multimodality and Inter-Entity Experience. *Frontiers in Public Health*, *14*, 1,603,238.
Human-delivered psychotherapy	Psychotherapy conducted by a licensed clinician who retains clinical responsibility and engages in a human therapeutic relationship.	In-person psychotherapy; clinician-led telepsychotherapy.	Fully automated systems; unsupervised self-help tools.	Grodniewicz, J. P., & Hohol, M. (2023). Waiting for a digital therapist: three challenges on the path to psychotherapy delivered by artificial intelligence. *Frontiers in Psychiatry*, *14*, 1,190,084.
Machine learning (ML)	A subset of AI that enables systems to learn patterns from data and improve performance on a task without being explicitly programmed with rule-based instructions.	Supervised learning algorithms (e.g., regression, classification models); Unsupervised learning (e.g., clustering, dimensionality reduction); Reinforcement learning systems; Deep learning models (e.g., neural networks).	Manually programmed rule-based systems without adaptive learning; Static statistical analyses that do not update or improve with data exposure; Simple deterministic algorithms without data-driven training.	Aafjes-van Doorn, K., Kamsteeg, C., Bate, J., & Aafjes, M. (2021). A scoping review of machine learning in psychotherapy research. *Psychotherapy Research*, *31*(1), 92–116.
Natural language processing (NLP)	A field of AI focused on enabling computers to process, analyze, understand, and generate human language in written or spoken form.	Text classification and sentiment analysis; Language generation (e.g., chatbots, text summarization); Speech recognition and speech-to-text systems; Machine translation; Named entity recognition and parsing.	Non-language-based data processing (e.g., image-only computer vision tasks); Purely rule-based string manipulation without linguistic analysis; Human-only language interpretation without computational processing.	Goldberg, S. B., Flemotomos, N., Martinez, V. R., Tanana, M. J., Kuo, P. B., Pace, B. T., … & Atkins, D. C. (2020). Machine learning and natural language processing in psychotherapy research: Alliance as example use case. *Journal of counseling psychology*, *67*(4), 438.
Retrieval Augmented Generation (RAG)	An AI architecture combining information retrieval with generative language models to ground outputs in external sources.	Guideline-anchored psychoeducation; source-linked responses.	Elimination of hallucinations; autonomous clinical reasoning.	Soman, G., Judy, M. V., & Abou, A. M. (2025). Human guided empathetic AI agent for mental health support leveraging reinforcement learning-enhanced retrieval-augmented generation. *Cognitive Systems Research*, *90*, 101,337.
Telepsychology / Telepsychotherapy / Teletherapy	Sessions delivered by a human clinician via digital communication technologies.	Video- or phone-based sessions delivered by licensed professionals.	Chatbots or AI systems acting autonomously.	Kocsis, B. J., & Yellowlees, P. (2018). Telepsychotherapy and the therapeutic relationship: Principles, advantages, and case examples. *Telemedicine and e-Health*, *24*(5), 329–334.

**Table 2:** Take-home messages

Domain	Take-home message
AI-supported supervision	AI shows potential as a supervision support tool, enhancing reflective practice, feedback structuring, and pattern detection across clinical work, while supervisory judgment, ethical responsibility, and final evaluative decisions remain firmly human-led.
Assessment, diagnosis, and treatment planning	AI shows the greatest promise as clinical decision support, assisting clinicians with synthesis and pattern recognition while responsibility and interpretation remain human.
Blended care models	The most robust integration pathway involves blended care, where digital tools enhance continuity and efficiency without replacing the therapist.
Chatbot-delivered interventions	Fully automated conversational agents may improve short-term symptoms and engagement, but should be conceptualized as self-help tools rather than psychotherapy.
Clinical risks and safety	Key risks include over-reliance, anthropomorphic attachment, insufficient crisis management, and data governance concerns, particularly for vulnerable populations.
Digital phenotyping	Passive digital data can support monitoring and research but requires careful ethical, interpretative, and privacy safeguards.
Public communication and terminology	Ambiguous labels (e.g., “AI therapist”, “virtual therapy”) should be avoided or explicitly defined to prevent miscalibrated trust and role confusion.
Reliability and hallucinations	LLMs can produce fluent but incorrect outputs; grounding methods such as RAG improve transparency but do not eliminate errors.
Research, practice, and the evidence–adoption gap	Invest in active-comparator RCTs and harm-focused outcomes; adopt AI as adjunctive with explicit safeguards; bridge the evidence–adoption gap through interim guidance, regulation, and clinician–patient dialogue.
Therapeutic alliance in digital contexts	Alliance can emerge in digital interventions, but its meaning and function differ depending on whether a human therapist is involved.

## Results

### Assessment and Treatment Planning

Initial evidence on the use of AI in clinical psychology and psychiatry suggests it can be effectively used for diagnosis and developing treatment planning [2, 3]. Indeed, the contribution that AI can make to identifying biomarkers to develop prognostic risk models for mental illnesses appears promising. For example, the integration of electroencephalography (EEG) data with deep learning techniques based on artificial neural networks seems capable of differentiating individuals with symptoms of depression from healthy individuals [9]. Regarding clinical reasoning, an exploratory study by D’Souza et al. [2] evaluated the adequacy of psychodynamic formulations generated by ChatGPT based on clinical vignettes, finding that GenAI can produce formulations that are generally consistent with the case material. However, the evidence is sometimes contradictory. For instance, Umutlu et al. [10] compared ChatGPT-4 with physicians on psychiatric case assessment. Compared to clinicians, ChatGPT-4 was effective at structuring cases, making differential diagnoses, and providing treatment suggestions, but it underperformed in in-depth interviewing, risk assessment, and side-effect monitoring.

## Psychotherapeutic Intervention

Recent evidence has explored the possibility of providing psychotherapy via a conversational agent or chatbot [11, 12]. This option offers several advantages in terms of accessibility and cost, making it a promising avenue for further investigation. GenAI-based therapists can retain user information over time and simulate consistent, non-judgmental listening, potentially enhancing engagement. Increasing personalization options and constant availability further expand their appeal as accessible support tools [13].

The first GenAI therapy systems (such as Woebot, Replika, Tess, Sara, and Wysa) appear to be effective in reducing anxiety and depressive symptoms in the short term [11, 14]. Studies conducted on Woebot showed that this virtual tool helps users identify their emotions and thought patterns, teaching them coping and anxiety-reduction strategies [12]. Woebot has been effective in assisting with relationships, grief, and depression, while Wysa has been effective for people suffering from stress, anxiety, and sleep loss. In comparison to Woebot, Wysa is better at providing specific exercises, which may be physical/behavioral or mental, in order to overcome difficult situations and depression [12].

Another very relevant recent Randomized Controlled Trial (RCT) study is that of Heinz et al. [11], who showed that one month of treatment with a GenAI chatbot for mental health treatment of 210 patients with depression, anxiety, or eating disorders led to a significant reduction in symptoms compared to those assigned to the waiting list control group, and that these results were maintained even after two months. In a similar RCT with a waitlist control group, Sharp and colleagues [15] reported comparable outcomes on 60 participants; additionally, participants in the chatbot group showed greater motivation to seek treatment, with 93% entering care within three months compared to 70% in the control group. However, in both RCTs, neither study staff nor participants were blinded, and the control conditions did not involve active human-delivered therapy, relying instead on waitlist or informational controls.

When comparing psychotherapy and GenAI chatbots, the data show that human-delivered therapy remains more effective [7]. In an RCT involving 104 women with anxiety disorders living in conflict zones, participants received either daily support from the Friend chatbot or intensive psychotherapy (three 60-minute sessions per week). Both groups demonstrated significant reductions in anxiety, although improvements were larger in the traditional therapy group (with human-certified psychologists). The chatbot offered meaningful benefits in settings with limited access to clinicians, but its emotional engagement and clinical impact were lower than human-delivered therapy [7]. Another two-arm trial compared an iCBT chatbot (Tinnibot) with a hybrid intervention combining Tinnibot and telepsychology in 30 adults with tinnitus [16]. Over the 8-week intervention and 2-month follow-up, both groups showed significant reductions in Tinnitus Functional Index scores, with a trend favoring the group that received telepsychology with the human psychologists.

## Clinical Risks

In light of the considerations outlined above, it is relevant to examine the key limitations and risks of GenAI-mediated therapy relative to human-delivered psychotherapy.

## Does the GenAI ChatbotTreatment Foster Autonomy?

Within the secure base established in human psychotherapy, clients gradually learn to recognize thoughts, emotions, and bodily sensations through attuned therapeutic feedback, fostering meaning-making and emotional awareness [17]. Over time, this process promotes autonomy, emotional tolerance, and compassion toward previously disowned or suppressed aspects of the self [18]. Unlike GenAI-based interactions, human therapy encourages a progressive reduction of dependence on the therapist as clients strengthen their capacity to understand themselves and the world [17].

GenAI interactions, by contrast, may activate the mesocorticolimbic reward circuitry involved in social reward and behavioral addiction. Neuroimaging studies show that online feedback and digital platform-related stimuli engage dopaminergic reward pathways in ways comparable to those observed in problematic social media and gaming use [19]. As a result, part of the satisfaction derived from GenAI interactions may reflect reward activation rather than therapeutic processing, increasing the risk of craving and compulsive use. Although some chatbots issue warnings about excessive engagement, these safeguards do not reliably prevent misuse [20]. Continuous 24/7 GenAI availability may further encourage a form of “fast-food psychotherapy,” characterized by immediacy and seduction rather than reflective depth. When clients seek solutions, GenAI may offer direct answers that risk reinforcing maladaptive patterns instead of fostering autonomy, insight, or relational change.

### Can GenAI Chatbot Treatments Provide Calibrated Mismatches?

Therapy requires more than simple mirroring, which risks reinforcing rigid client patterns. To avoid collusion, therapists introduce calibrated mismatches—strategic divergences that offer alternative interpretations and allow clients to see their worldview as one perspective among others [21]. Psychotherapists continuously decide when to mirror, reframe, or introduce dissonant alternatives and perturbations to patients’ rigid schemas, guided by bodily resonance, silence, or paradoxical interventions [17].

Conversely, a GenAI chatbot, unless it is trained with an adequate degree of clinical sensitivity, tends to operate through a compliant and seductive logic. By collusively mirroring users, these aspects may deprive clients of the essential human mismatch that fosters insight and self-awareness. GenAI therapy applications often emphasize self-soothing techniques such as mindfulness exercises, breathing techniques, and guided reflections. While these strategies are usually appreciated, participants felt the apps might fail to encourage active behavioral change in a deep, transformative way [6]. Even if future GenAI could generate attuned mismatches or perturbations, the absence of a lived identity and personal perspective may limit the extent to which such interventions feel authentic, timely, or therapeutically grounded.

## Users’ Profiles and GenAI: Who Is It Most Effective For?

GenAI-led therapy success depends on technological, socio-cultural, and individual synergy. Generally, men and early adopters show higher trust. Recent data suggests that younger users and those with lower educational levels are more accepting, likely due to daily technological familiarity [22, 23]. This shifts the focus not so much toward how authentic the GenAI’s curiosity might be, but toward how much it is perceived as such by users according to their expectations [24]. Viewing GenAI with uncritical optimism risks creating an illusion of care, as its functioning is shaped by market-driven rather than clinical logic. These concerns have prompted international governance efforts, including WHO [25]. Research shows that messages are perceived as more reliable when users believe the source is human [8], underscoring the need for clear framing: users must understand when they are interacting with a virtual system generating plausible outputs, not an intentional agent [26]. This human-like perception often leads to quasi-relationships where users feel comfortable making unexpected personal disclosures, which in turn raises critical ethical and legal questions. Specifically, disclosures of crimes to chatbots challenge whether technology providers share the same reporting duties as healthcare professionals. This creates a conflict between the duty to protect potential victims (justice) and the need to avoid mistaken reports that undermine user trust (non-maleficence), requiring a careful balance of ethical principles across different jurisdictions [20].

A further safety concern is that excessive GenAI interaction may exacerbate psychotic symptoms. GenAI’s sycophantic tendency can act to reinforce delusional ideation rather than challenge it or trigger manic symptoms [27, 28]. In Heinz et al.’s study [11], human intervention was required multiple times for suicidal ideation reported to a chatbot. Despite advances in safety tuning, increasing anthropomorphization heightens these risks. Vulnerable populations, such as minors or individuals with impaired reality testing, may mistake algorithmic reinforcement for genuine care. While GenAI can display pseudo-empathy, it lacks moral responsibility and authentic relational involvement [7].

## Future Integration

### Strengthening Resources and Monitoring between Sessions

For some clients carefully selected by the therapist, it may be useful to have an AI tool that tracks their impressions at the end of the session, or that provides a space for reflection to be shared with the therapist. In this space, the therapist could write down any considerations, exercises, or emotional recognition tools that they want to share with the patient [29]. At certain points in therapy, this shared space may reinforce work conducted between sessions, supporting a gradual process of empowerment and self-efficacy. The presence of this AI support, always supervised by a human therapist, could be valuable, for example, in high-risk situations that require monitoring the client to prevent self-harm [7, 28].

### GenAI in Clinical Training and Supervision

GenAI-based clinical training for psychotherapy students could be implemented under the supervision of experienced clinicians. Through interactions with virtual patients presenting diverse psychopathological profiles, students could practice assessment and intervention skills in a protected learning environment [30]. Virtual-patient simulations based on Large Language Models (LLMs) also provide a safe space for trainees to practice interviewing skills and receive structured feedback before working with real clients [31]. However, ongoing supervision remains essential to help trainees recognize errors, reflect on their interventions, and remain aware of the limitations of simulated clinical encounters.

In supervision with real patients, GenAI can provide process indicators by analyzing sessions longitudinally, offering feedback on symptom trends, alliance, and patterns to identify stagnation [32]. LLMs can also assist in generating formulations or organizing clinical data. However, lacking a grasp of transferential dynamics, GenAI outputs must remain mere hypotheses to stimulate reflection, ensuring clinician autonomy and ethics [33].

### Can GenAI Chatbot Treatments Reduce Social Avoidance? Potential and Risks

Severely lonely individuals often feel more comfortable sharing intimate concerns with GenAI than those less lonely, with social loneliness correlating to higher GenAI acceptance [34, 35]. For individuals experiencing pervasive shame or traumatic invalidation (e.g., those exhibiting hikikomori symptoms) [36–38], GenAI can be perceived as a safer alternative to human relationships perceived as dangerous.

For vulnerable users, GenAI’s non-judgmental nature may provide relief from the evaluating gaze [13]. A cautiously integrated, blended approach could be explored to investigate whether GenAI can serve as a temporary reflective diary for shame-laden material, later processed in human therapy. GenAI use should therefore remain regulated and time-limited, integrating GenAI capabilities with the transformative potential of the human connection [39].

### Exploring Clients’ Possible Self-Versions through Immersive AI

A growing body of research on clinical applications of virtual reality shows that immersive environments can foster self-compassion, reduce self-criticism, and enhance self-protection through perspective-shifting experiences and the embodiment of different self-versions [40, 41]. Building on these premises, future integrations between AI and therapists could specifically focus on parts work. For instance, a recent project, “Introspecta VR,” combines virtual reality and AI to help users explore their present and future self through visible, narratable identity configurations [41]. In this project, patients choose scenarios representing specific self-aspects, which are then modified by AI through prompts to increase evocative power. This approach facilitates work between individual parts, making internal conflicts and blockages more explicit.

## Discussion

### Assessment and Treatment Planning Take-Away Message

Assessment and treatment planning are two areas in which AI appears particularly promising. Initial evidence suggests that AI-based tools can assist diagnostic reasoning by integrating heterogeneous information (e.g., symptom profiles, clinical history, digital measures, and neurophysiological data), potentially saving time and improving assessment reliability [9]. In addition, generative models may facilitate therapeutic planning by organizing complex case material, producing structured case formulations, and proposing treatment plans that clinicians can refine [2, 3]. Nevertheless, AI applications should be framed as decision support rather than autonomous decision-making [10]. Future implementation of AI to support assessment and treatment plan formulation will depend on robust external validation across settings and populations, transparency about model limitations, and safeguards against privacy breaches.

### Psychotherapy Take-Away Message

Symptom reduction observed in GenAI-supported RCTs may reflect GenAI’s ability to generate emotionally supportive, personalized communicative exchanges. Through pseudo-empathic responses and partial mirroring, users can experience a sense of connection that facilitates self-expression and a preliminary emotional recognition, potentially explaining early improvements reported in some studies [14].

Despite substantial investment in GenAI development and increasingly refined emotional detection capabilities [6], technological sophistication should not be conflated with genuine therapeutic synchrony. It might remain questionable whether such systems can truly simulate the depth of a relational encounter or replicate the authentic relational climate of human connection. Virtual interactions, though technically advanced, may lack the profound intersubjective grounding and the full weight of corporeal presence that are generally considered fundamental to making human therapy credible, impactful, and transformative [5, 17]. These elements of physical and emotional co-presence form the backbone of the therapeutic alliance, a dimension that current technology can mimic but perhaps not fully embody [26]. Even if future GenAI systems generate forms of “resonance,” these will remain qualitatively different from human presence. For these reasons, GenAI is best conceptualized as a supportive or integrative tool rather than a replacement for human psychotherapy.

### Risks Take-Away Message

Unmonitored use of GenAI in psychotherapy could compromise clients’ autonomy and confirm rigid schemas about the self, others, and the world, risking collusion with the client without fostering awareness, autonomy, or different relationship skills [28]. Notably, GenAI may become a belief confirmer, reinforcing delusional ideation, manic states, or aggressive attitudes [11]. These risks are amplified by the fact that real-world adoption is currently outpacing controlled evidence, making the definition of interim safeguards a clinical priority. An additional key element concerns the nature of the datasets used to train general-purpose LLMs and many current GenAI systems. Most models are trained on large-scale internet-derived corpora rather than clinically curated psychotherapy datasets, raising concerns regarding representativeness, cultural bias, diagnostic validity, and contextual reliability. As a result, outputs may reflect hidden biases, inaccuracies, stereotyped assumptions, or clinically inappropriate associations that are difficult to detect in real time [42]. Furthermore, many GenAI systems currently lack robust external validation across diverse clinical populations, languages, and treatment contexts [42]. These limitations increase the risk of both known and unforeseen errors, particularly when systems are applied to vulnerable populations or high-stakes clinical decisions [43, 44]. For these reasons, GenAI outputs should be interpreted cautiously and always remain subject to clinician oversight and contextual clinical judgment.

### Future Integration Take-Away Message

Future integration should aim to support the therapist and protect the human nature of psychotherapy, capitalizing on GenAI’s strengths and capacities. Regarding psychotherapy practice, AI can help monitor variables between sessions, support a shared reflective diary, or prompt exercises that consolidate session work, and prepare material for discussion. In training and supervision, AI-generated structured feedback can improve clinicians’ interviewing, case formulation, and technique skills. Within this perspective, AI integration needs clear rules on goals and boundaries and should remain embedded in an active human therapeutic alliance [45].

### Recommendations for Research, Clinical Practice, and Bridging the Evidence–Adoption Gap

#### Research Priorities

The current evidence base for GenAI in mental health remains preliminary. Future research should prioritize: (i) RCTs with active human-delivered comparators rather than waitlist or information-only controls, and with assessor blinding where feasible; (ii) long-term follow-up beyond 8–12 weeks to evaluate symptom durability, dependency, and iatrogenic effects; (iii) harm-focused endpoints, including reinforcement of delusional or manic ideation, fostering of compulsive use, mishandling of suicidal disclosures, and erosion of help-seeking from human professionals; (iv) heterogeneity-of-treatment-effect analyses that identify which users benefit (e.g., mild-to-moderate symptoms, motivational support) and which may be harmed (e.g., psychosis-spectrum, severe loneliness, minors); (v) process-level studies measuring therapeutic alliance, autonomy, and calibrated mismatches; (vi) independent external validation of diagnostic and prognostic models across cultures, languages, and clinical settings; and (vii) implementation and post-market surveillance studies that capture real-world use rather than ideal-condition trials.

#### Clinical and Organizational Implementation Now

 Until stronger evidence is available, we recommend that clinicians and care organizations adopt a conservative approach, as GenAI tools should support, not replace, human-delivered care. Concrete guidelines include: (a) explicit informed consent and disclosure that the user is interacting with a non-sentient system; (b) risk stratification at intake, excluding or carefully monitoring high-risk users (e.g., individuals with psychosis-spectrum conditions, active suicidality, or severe dissociation); (c) time-limited and goal-bounded use (e.g., between-session psychoeducation, mood tracking, behavioral rehearsal); (d) clear procedures to involve a human clinician in case of emergency; (e) clinician training in critical appraisal of GenAI outputs, to counter automation bias and cognitive offloading; (f) data governance standards consistent with healthcare privacy frameworks; and (g) organizational accountability, with named clinical supervisors responsible for GenAI-assisted care pathways.

#### Navigating the Tension between Evidence and Adoption

The pace at which patients and providers are already adopting general-purpose chatbots for mental-health support clearly outstrips the available controlled evidence. Therefore, we recommend the following strategies: (1) professional societies should release provisional practice guidelines, to be revised iteratively as new evidence becomes available; (2) regulators (e.g., WHO, EU AI Act, FDA) should require transparency and adverse-event reporting for tools marketed for mental-health use; (3) clinicians should proactively ask patients about their AI use and integrate it into the case formulation; and (4) psychoeducation should clarify what GenAI can and cannot do, especially regarding empathy, confidentiality, and crisis response. The goal is not to slow innovation but to align it with the relational and ethical framework of mental-health practice. Ultimately, AI is a powerful tool that may illuminate patterns throughout the therapeutic journey and open new possibilities, but the delicate work of holding another mind in mind must remain a fundamentally human task.

## Key References

- Galido et al. [3]:
  - ○ This case study indicates that ChatGPT is able to align treatment-resistant schizophrenia management with current medical standards, suggesting its potential as a supportive tool for organizing data within clinical practice.
- Kuo et al. [32]:
  - ○ This study utilized a transformer-based NLP model (RoBERTa) to predict client symptoms from 2,630 sessions, proving far more effective than traditional word-counting models. By analyzing the contextual relationship between phrases rather than isolated keywords, the model accurately interprets complex clinical nuances, such as whether a desire to terminate therapy reflects progress or dropout. These findings establish advanced linguistic analysis as a reliable, scalable tool for monitoring therapeutic outcomes.
- Heinz et al. [11]:
  - ○ This RCT suggests that an expert-tuned GenAI chatbot can reduce clinical symptoms of depression and anxiety, showing high user engagement and strong therapeutic alliance. It validates GenAI as a feasible, personalized first-line intervention capable of delivering measurable clinical improvements.
- Sharp et al.[15]:
  - ○ A Chatbot Single-Session Intervention acted as an effective, scalable early intervention for individuals on eating disorder waitlists, reducing pathology, depression, and anxiety while increasing treatment uptake compared to standard web-based information.
- Spytska [7]:
  - ○ This randomized controlled trial on 104 women in war zones found that while both interventions significantly reduced anxiety, traditional psychotherapy outperformed the "Friend" chatbot, suggesting that while GenAI is a vital scalable tool for immediate crisis support, it currently lacks the emotional depth and efficacy of human-led therapy.
- Yirmiya & Fonagy [26]:
  - ○ This paper argues that GenAI lacks the genuine emotional presence and reciprocal intentionality necessary to foster authentic therapeutic trust. The authors suggest that GenAI’s role should be strictly adjunctive, enhancing the therapist’s capacity within a supervised clinical framework.
- Østergaard [27]: 
  - ○ Chatbots may reinforce manic states by providing constant, uncritical validation that fuels the user’s cognitive distortions. This interaction highlights the risk of "collusion" between GenAI and the patient’s pathology, confirming the need for strict clinical oversight.
- Cioffi et al. [33]:
  -  ○ Trainees evaluated feedback on a clinical case provided by untrained GenAI, pre-trained GenAI, and a human supervisor. The findings suggest the potential of calibrated GenAI as a complementary tool in clinical supervision.
- Antichi et al. [41]:
  - ○ Introspect VR utilizes a triadic framework of real-time, AI-generated "skyboxes" to create immersive, 360° metaphorical environments that allow participants to visually explore their personal identity development through personalized landscapes.

## Data Availability

No datasets were generated or analysed during the current study.
